# Genotypic and Environmental Effects on the Volatile Chemotype of *Valeriana jatamansi* Jones

**DOI:** 10.3389/fpls.2018.01003

**Published:** 2018-07-10

**Authors:** Xingchao He, Shiyu Wang, Jiayi Shi, Zhonglin Sun, Zhentian Lei, Zili Yin, Zigang Qian, Huiru Tang, Hui Xie

**Affiliations:** ^1^School of Pharmacy, Fudan University, Shanghai, China; ^2^State Key Laboratory of Genetic Engineering, Zhongshan Hospital and School of Life Sciences, Collaborative Innovation Centre for Genetics and Development, Shanghai International Centre for Molecular Phenomics, Human Phenome Institute, Fudan University, Shanghai, China; ^3^School of Traditional Chinese Medicine, Yunnan University of TCM, Kunming, China; ^4^Metabolomics Center, University of Missouri, Columbia, SC, United States; ^5^School of Chinese Ethnic Medicine, Yunnan University of TCM, Kunming, China

**Keywords:** Valerianae Jatamansi Rhizoma et Radix, *Valeriana jatamansi*, genotype, chemotype, volatile profile, wild population, common-garden sample

## Abstract

*Valeriana jatamansi* Jones is an aromatic medicinal herb and important alternative to *V. officinalis*, which is utilized for medicinal purposes in China and India and also as spices in India. Bioactive ingredients of *V. jatamansi* vary in different regions. However, no information is currently available on influence of genotype and environmental factors in the volatile compounds, especially when germplasms and planting locations need to be selected. Based on the results of SNP and volatile constituents from GC-MS analysis, this study found various genotypes and chemotypes of *V. jatamansi* for wild plants from seven regions in China and common-garden samples; correlations between genotype and chemotype were revealed for the plants. Two distinct populations (PX, FY) were distinguishable from five others (GJ, YL, SY, DD, DY) according to their genotypes and volatile profiles, the consistency of which was observed showing that genotype could significantly influence chemotype. Wild populations and common-garden samples were also separated in their volatile profiles, demonstrating that environmental factors strongly affected their chemotypes. Compounds contributing to the discrimination were identified as discriminatory compounds. This investigation has explored and provided essential information concerning the correlation between genotype and chemotype as well as environmental factors and chemotype of *V. jatamansi* in some regions of China. Feasible plantation and conservation strategies of *V. jatamansi* could be further explored based on these results.

## Introduction

Biogenesis of essential oil occurs widely across the plant kingdom and is important for plant physiology in terms of metabolism and preset developmental differentiation programme of the synthesizing tissue (Chappell, 1995; Sangwan et al., 2001). As an essential part of plant metabolism, the biosynthesis of essential oil may depend on both the genetic backgrounds and environmental effects (Chappell, 1995; Sangwan et al., 2001). Commercially, these volatile plant metabolites have already found extensive cosmetic and therapeutic applications (Sangwan et al., 2001).

*Valeriana jatamansi* Jones is a valuable plant species with volatile components utilized as a herbal medicine. Valerianae Jatamansi Rhizoma et Radix is indexed in Chinese Pharmacopeia (Part 1) in the 1977, 2010, and 2015 editions as a traditional Chinese medicine for gastrointestinal diseases and anxiety. It is prepared from naturally dried rhizome and root of perennial herb *V. jatamansi* Jones, which is widely distributed throughout temperate Himalayan region and southwestern areas of China (Ming et al., 1997; Jugran et al., 2013). Many phytochemicals including iridoids, sesquiterpenoids, and essential oils have already been identified from this species (Ming et al., 1997; Bhatt et al., 2012; Li et al., 2013). Among them, essential oils commonly used as spices in India had both antioxidant and insecticidal activities whereas iridoids and sesquiterpenoids showed moderate neuroprotective effects and inhibitory activity on acetylcholinesterase (Rana and Sharma, 2000; Xu et al., 2011; Dong et al., 2015), respectively.

For these traditional and newly found pharmaceutical purposes, this medicine's consumption is increasing in these years and the distribution range of crude drugs is expanding. The essential oil composition was explored and was considered to be largely influenced by plant's location of growing (Verma et al., 2011), while some non-volatile components such as the valepotriates are supposed to be similar (Wang et al., 2017). Chinese Pharmacopeia (2015 edition) requires assessments of size, exterior character, water content, total ash, acid-insoluble ash, total extractives, and microscope characteristics together with the TLC profiles of valepotriane (C_22_H_30_O_8_) and acetylvalepotriane (C_24_H_32_O_10_). However, the quality control of this medicinal herb and resource recognition from different areas remain difficult if based solely on the above descriptions. Selection of good germplasms and good agricultural practice (GAP) of *V. jatamansi* is required, and planting locations is ought to be selected properly. It is well-documented that chemical phenotypes (chemotypes) of several herbal medicines depends on the environment they grow (Wang et al., 2004; Dai et al., 2010a,b). However, whether *V. jatamansi* possesses different genotypes and how these genotypes and environmental factors affect its chemotype remain to be clarified. To the best of our knowledge, few researches about volatile constituents of *V. jatamansi* have been published so far.

To address these questions, in current study, type IIB endonucleases restriction-site associated DNA (2b-RAD) approach (Wang et al., 2012) was used to identify the genotypic characteristics for seven wild populations of *V. Jatamansi* from China. The head-space of SHS-GC-MS analysis would cover all volatile components including not only essential oils but also components with low boiling points, water-soluble volatile components and/or oxidation-prone components (Molina-Calle et al., 2017). Therefore, the composition of volatile compounds was also analyzed as plant chemotype here for all wild populations together with the common-garden samples originating from them. Dependence of the chemotypes on the genotypes and environmental factors was further evaluated for Valerianae Jatamansi Rhizoma et Radix.

## Materials and methods

### Plant materials

Although *V. jatamansi* is widely distributed throughout temperate Himalayan region and southwestern areas of China, the origin plants of Valerianae jatamansi Rhizoma et Radix used for medicinal purposes are traditionally collected in Yunnan and Guizhou provinces. To clarify whether different genotypes and chemotypes were present, seven wild populations of *V. jatamansi* were collected from seven different locations in Yunnan and Guizhou provinces to cover traditional sampling areas plus environmental locations. These included samples from Gejiu (GJ) and Fuyuan (FY) in Yunnan Province, Leishan Yongle (YL), Leishan Dadi (DD), Suiyang (SY), Panxian (PX), and Duyun (DY) in Guizhou Province (Table 1, Supplemental Figure 1).

**Table 1:** Information of selected regions plant were sampled.

**Population**	**Voucher**	**Season**	**Location**	**Longitude**	**Latitude**	**Altitude/m**	**Slope**	**Biotope**
PX	WT01	Oct. 2015	Panxian, Guizhou	104°24′	25°40′	1946	North	Mingled forest
FY	FY01	Oct. 2015	Fuyuan, Yunnan	104°13′	25°34′	1939	North	Farmland margins
GJ	GJ01	Aug. 2015	Gejiu, Yunnan	103°12′	23°23′	2530	Southeast	Hillside meadows
DD	LS0202	Sep. 2015	Dadi, Leishan, Guizhou	108°08′	26°08′	1381	South	Hillside meadows
DY	FY01	Oct. 2015	Duyun, Guizhou	107°36′	26°18′	918	Northwest	Mingled forest
YL	LS0102	Sep. 2015	Yongle, Leishan, Guizhou	108°09′	26°11′	1056	East	Farmland margin
SY	SY02	Sep. 2015	Suiyang, Guizhou	107°13′	27°59′	1030	Northwest	Farmland margin

In each population, the leaves of 10 plants were collected randomly with spacing greater than 5 m, dried with allochroic silica gel and preserved in normal temperature for genetic analysis. The plant was identified by Professor Zigang Qian, Yunnan University of Traditional Chinese Medicines and a voucher specimen for all the plant material was deposited in The Chinese Medicine Specimen Museum of Yunnan University of Traditional Chinese Medicines for further reference (Table 1).

To avoid seasonal effects on the plant metabolite composition (Xiao et al., 2008), we collected wild samples within a shortest period (practically possible) in autumn according to accumulated experiences of Traditional Chinese Medicine. To take the effects of environmental factors (e.g., altitude, temperature, sunshine, and humidity) into consideration, we also collected the common-garden samples (Yunnan Longfar Pharmaceutical Co. LTD) within 2 days during the same season. Common-garden samples were collected 1 year after transplantation using the same approach as for the corresponding wild populations. The wild plants were also transplanted to the Planting Base in Kunming Unity Township.

For volatile constituent analysis, the rhizomes and roots were removed from the same plants, dried and preserved in the same way described above. Samples were cleaned with brush, cut into pieces with a pair of scissors and ground into powder using SKSI tissue lyser (BiHeng Biotechnology Inc.) at 60 Hz for 30 s with 3 cycles totally. Ground samples were sieved through a 40 mesh sieve and then stored in sample bottles with good sealing property at 4°C before further analysis.

### Genetic analysis based on 2b-RAD approach

DNA was extracted by means of AxyPrep^TM^ Multisource Genomic DNA Miniprep Kit 50-prep (AP-MN-BT-GDNA-50G, Axygen, USA). Mass and concentration of DNA were measured using nucleic acid and protein analyzer.

Sequencing libraries with tags from 70 samples of *V. jatamansi* were built based on 2b-RAD approach (Wang et al., 2012). 5′-NNN-3′ was used to establish libraries for all samples. Paired-end sequencing was conducted for qualified libraries on Hiseq Xten platform.

Tags of individual samples were clustered into allele clusters and mismatch was not allowed. Allele clusters were combined into locus cluster to form reference sequence when two mismatches were allowed. Low-coverage locus cluster formed by sequencing error and high-coverage locus cluster structured by repetitive sequences were excluded based on improved maximum likelihood (iML, whole-genome *de novo* SNP genotyping method based on mixed Possion distribution model). Threshold was obtained from distribution model of tag numbers. Finally 300,926 tags with BsaXI enzyme cutting sites were obtained.

Original sequences were formed by base calling (raw reads) and were filtered by excluding sequences including N bases and reads with flanking sequences as well as low quality (more than 20% bases with quality less than 20) to get clean data.

### Volatile constituent analysis

#### SHS-GC-MS analysis

Samples were brought to room temperature. Three replicates of powdered samples (about 0.1000–0.1010 g) were put into three different glass vials (20 mL) then sealed with screw-caps having PTFE/silicone septa (17-02-1417-TF, SHIMADZU).

Static headspace equilibration was conducted at 140°C for 20 min with 400 rpm shaking. Then 2.00 mL of headspace gas was injected into injection port and analyzed using QP2010 Ultra Gas Chromatography-Mass Spectrometer (SHIMADZU, Japan) equipped with a Thermo Fisher non-polar TG-5MS column (P/N 26098–2970, 0.32 mm × 30 m × 1 μm); inlet temperature was set to 250°C with split ratio of10:1, helium gas as carrier gas (with a constant flow of 2 mL/min). Mass spectra were recorded with an electron ionization mode (70 eV), a scan range of m/z 50–1,000, ion source temperature of 250°C, transfer line temperature of 280°C, and solvent delay time of 2 min. Under the optimum conditions above, the relative standard deviations (RSD) of all compounds were lower than 20%.

#### Identification of volatile compounds

The GC-MS raw data were extracted and analyzed using GCMS Solution (Version 2.70). Areas of volatile compounds were calculated automatically with the same integration parameters (slope: 1,000/min, peak width: 2 s, drift: 0 min, smoothing time: 2 s, smoothing width: 2 s). Compounds were identified by automatic retrieval of the mass spectra (NIST 14 and NIST 14s), assisted by comparison between retention indexes from libraries and calculated ones based on experimental retention time of n-alkanes.

#### Statistical analysis

For genetic analysis, each genotype with markers was assembled head to tail and missing sites were replaced by “–.” Neighbor-joining tree and maximum-likelihood tree were structured using treebest (Version 1.9.2) and MEGA6. Two different analytical methods were employed here to ensure that such clustering were method independent since no single perfect method is available for the time being. Reliability was tested by bootstrap with 1,000 repeats. Genetic differentiation and diversity were presented using Genepop (version 4.2.2).

The compounds, which cover less than 80% of volatile profile of a single population or have signal-to-noise value lower than 10, were excluded from further analysis. Data, based on the areas of volatile compounds, was normalized by mass and scaled using Pareto mode using MateboAnalyst 3.0. Principal Component Analysis (PCA) was conducted for wild populations and common-garden samples separately, where samples outside 95% confidence intervals were excluded.

Clustering analysis was made using MateboAnalyst 3.0 with the distance measurement of Pearson and the Clustering Algorithm of Average. Results were further confirmed with Orthogonal Partial Least Squares-Discriminant Analysis (OPLS-DA) using SIMCA 14.1. S-plots and loadings plots were made to identify discriminatory compounds, verified through Variable Importance in the Projection (VIP) values automatically.

## Results and discussion

### Genotypic diversity and differentiation for *V. jatamansi*

F-statistics (Fst) was obtained for all populations and ranged from 0.0226 to 0.2344 (Table 2). Relatively high population differentiation was observed between PX, FY, and other populations with Fst in the range of 0.15–0.25. In contrast, medium inter-group differentiation was observed for PX and FY, for GJ and YL, for GJ and SY with Fst only slightly higher than 0.0500. Other populations shared lower differentiation with each other.

**Table 2:** Genetic differentiation (Fst).

**Population**	**GJ**	**YL**	**DD**	**SY**	**FY**	**PX**
YL	0.0536					
DD	0.0437	0.0226				
SY	0.0558	0.0369	0.0338			
FY	0.2344	0.2310	0.2257	0.2275		
PX	0.2145	0.2109	0.2049	0.2093	0.0607	
DY	0.0439	0.0388	0.0243	0.0376	0.2302	0.2093

Polymorphism Information Content (PIC), Expected Heterozygosity (He), and Observed Heterozygosity (Ho) were also calculated based on the result of SNP (Table 3). Apparently populations of PX and FY had higher PIC, He, and Ho than other populations. This demonstrated they were supposed to own higher genetic diversities.

**Table 3:** Genetic diversity.

**Population**	**He**	**Ho**	**PIC**
GJ	0.3650	0.5758	0.2887
YL	0.3691	0.5855	0.2908
DD	0.3531	0.5476	0.2800
SY	0.3661	0.5777	0.2888
FY	0.4049	0.6846	0.3152
PX	0.4080	0.6804	0.3179
DY	0.3499	0.5379	0.2778

**Figure 1:**
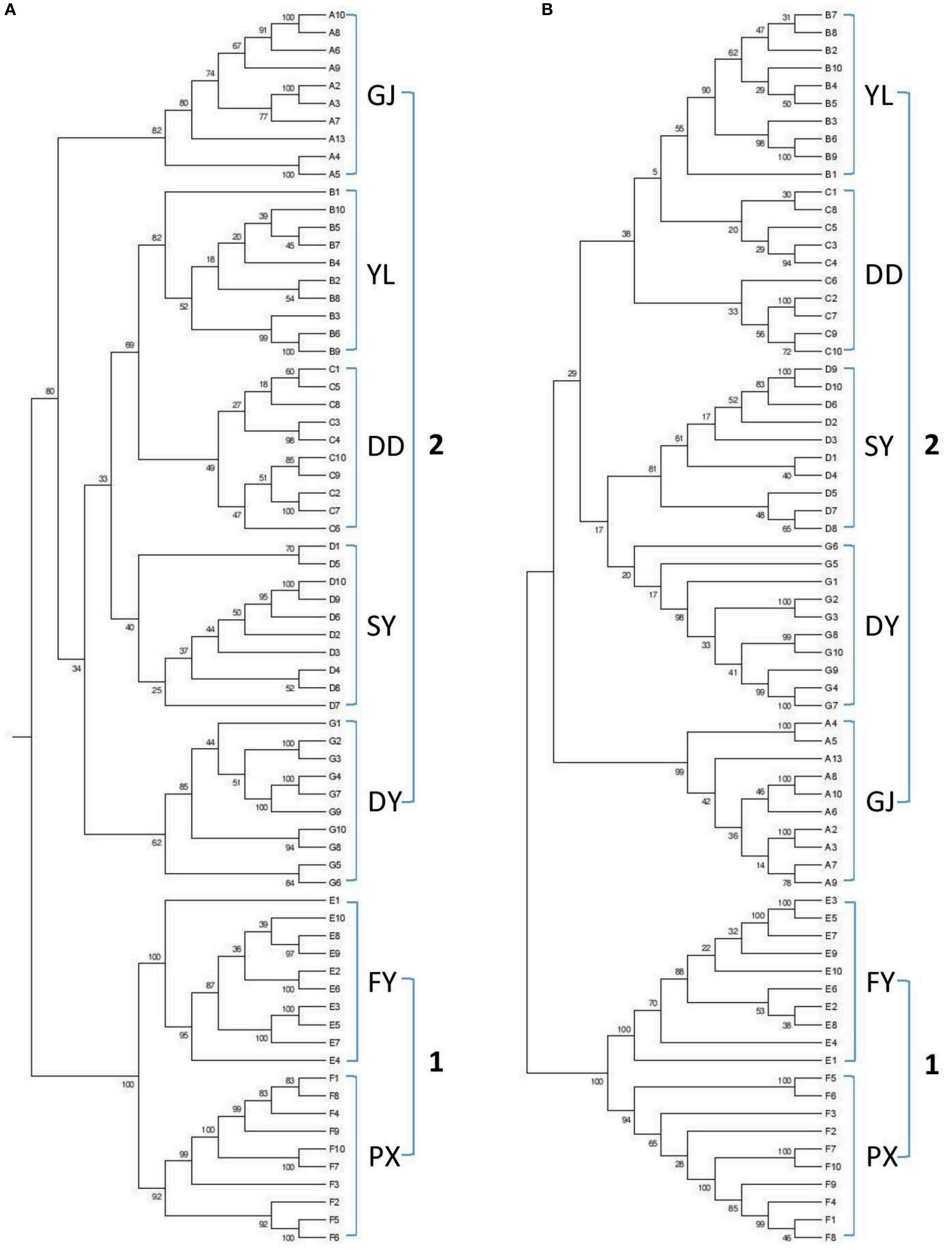
Dendrograms based on genetic analysis (A: GJ, B: YL, C: DD, D: SY, E: FY, F: PX, G: DY). **(A)** Neighbor-joining tree, **(B)** Maximum-Likelihood tree.

Dendrograms were generated based on DNA sequences for (wild) populations from various regions using reduced-representation sequencing 2b-RAD approach. Two analyses were also conducted from both Neighbor-joining tree (Figure 1A) and Maximum-Likelihood tree (Figure 1B). Results from both analyses clearly showed genetic similarity for populations PX and FY (known as genotype 1 or G1 in the following discussions) and similarity for the rest populations (genotype 2 or G2).

### Influence of genotype and environment on chemotype of *V. jatamansi*

Chemotypes of *V. jatamansi* were evaluated by measuring the composition of volatile compounds with GC-MS method in the aforementioned wild and common garden populations. Sixty-four such volatile compounds were detected and assigned by comparing their retention indices and mass spectral features with the NIST database (Supplemental Table 1). It is apparent that C10 (3-methylvaleric acid), C13 (sabinene), C43 (terpinylacetate), C49 (α-santalene), C50 (β-caryophyllene), C53 (eudesma-3,7(11)-diene), and C58 (α-bulnesene) were abundant volatile compounds in these plants (Supplemental Table 2). It is also interesting to note that many of these volatile metabolites are biosynthetic products terpenoid pathway from geranyl diphosphate and (2E,6E)-farnesyl diphosphate. For instance, C11 (camphene), C13 (sabinene), C14 (β-pinene), C22 (4-R-limonene), and C28 (terpinolene) are metabolites from geranyl diphosphate; C46 (β-elemene), C50 (β-caryophyllene), C52 (S-β-bisabolene), C54 (α-humulene), and C55 (α-patchoulene) are metabolites from (2E,6E)-farnesyl diphosphate (https://www.plantcyc.org/). These 64 compounds are here collectively employed to represent the sample chemotypes, reflecting multiple pathways in the secondary metabolism of this plant.

**Figure 2:**
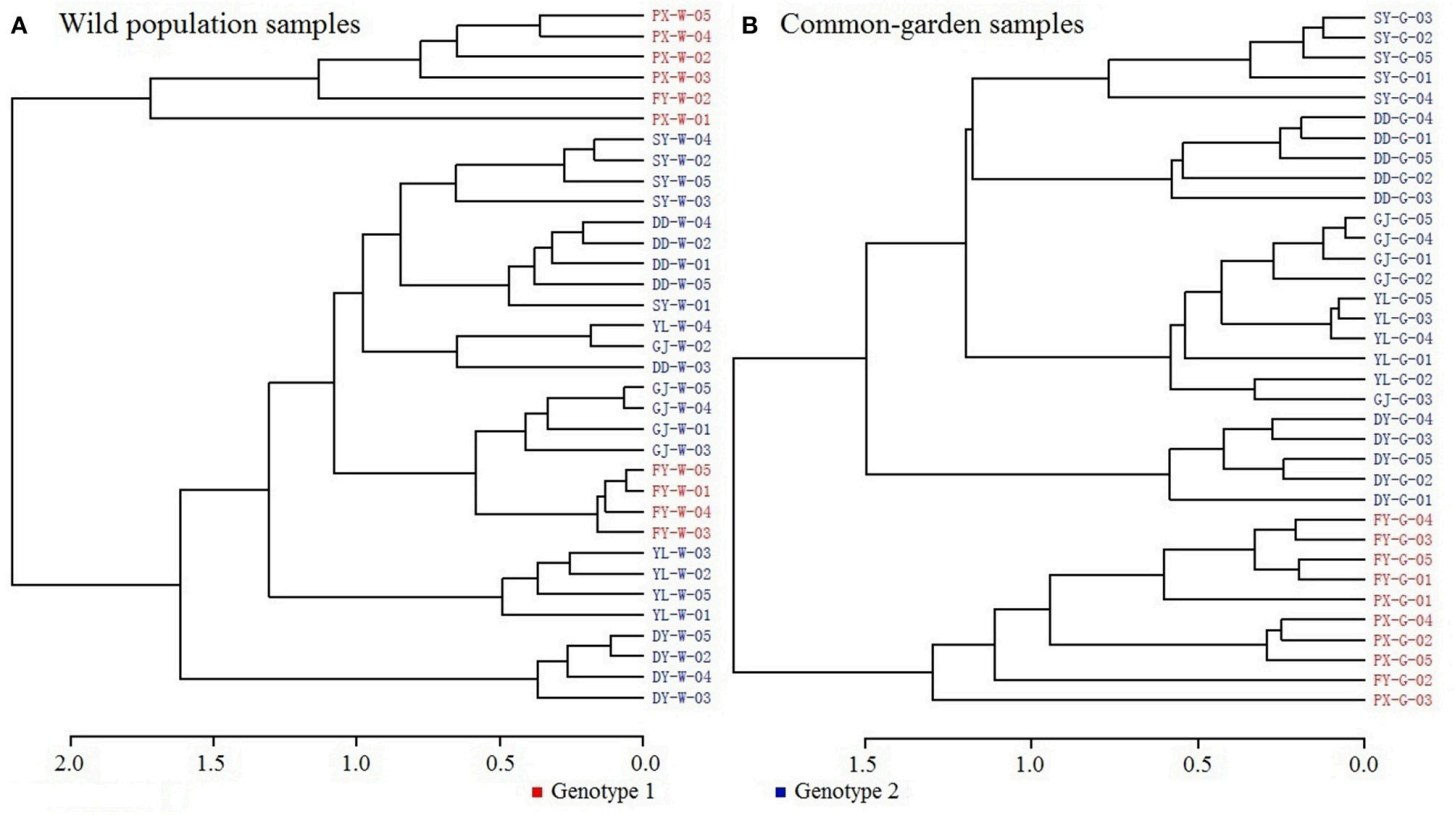
Dendrograms of volatile constituent analysis. **(A)** Wild population samples, **(B)** Common-garden samples.

Clustering dendrograms from the volatile constituents (Figure 2A) showed that amongst the wild seven populations, PX was outstandingly different from the rest six populations in terms of chemotypes. This apparently differed from the above genotyping results, where PX and FY clustered but differentiated from the other five populations. This indicates that environmental factors play important roles in establishing chemotypic characteristics. Such notion was further supported by the results that the chemotypes of common garden samples for PX and FY had greater similarities (with closer clustering) than the other five populations (Figure 2B) as shown from the genotypic analysis. This strongly suggests that both genotypes and environmental factors affect the chemotypic characteristics of plants.

**Figure 3:**
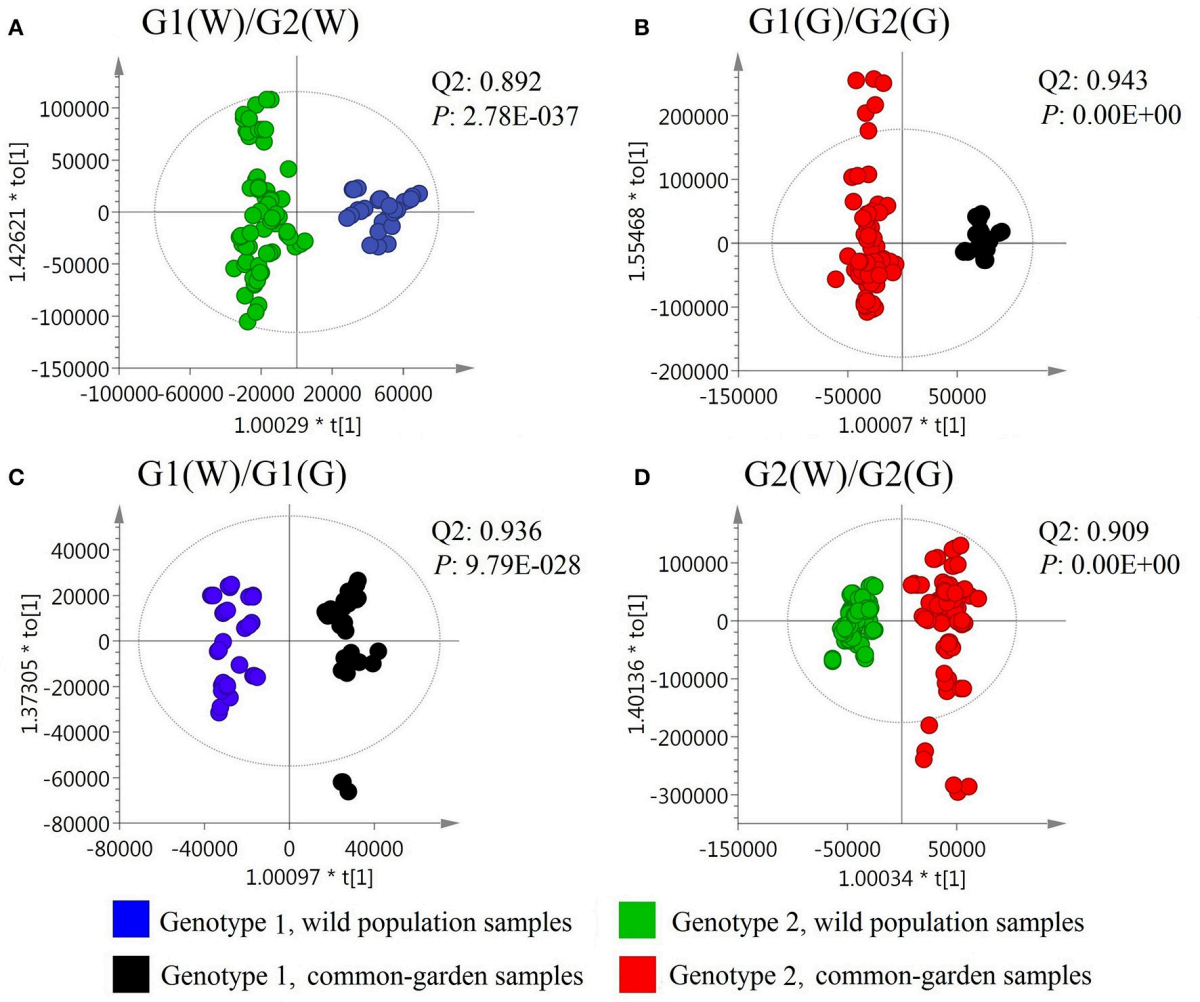
OPLS-DA results for the volatile compound composition.

To obtain further details for such chemotypic differences, OPLS-DA of volatile compound composition (chemotypes) was performed for wild and common garden samples in genotype 1 (G1) and 2 (G2) populations. The results showed that G1 and G2 had outstanding chemotypic differences for both wild and common garden samples with Q^2^ greater than 0.89 whilst *p*-values smaller than 2.8e–37 in both cases (Figures 3A,B). Furthermore, both G1 and G2 showed significant chemotypic differences between the wild and common garden samples with Q^2^ greater than 0.9 whilst *p*-values smaller than 9.8e-28 in both cases (Figures 3C,D).

**Figure 4:**
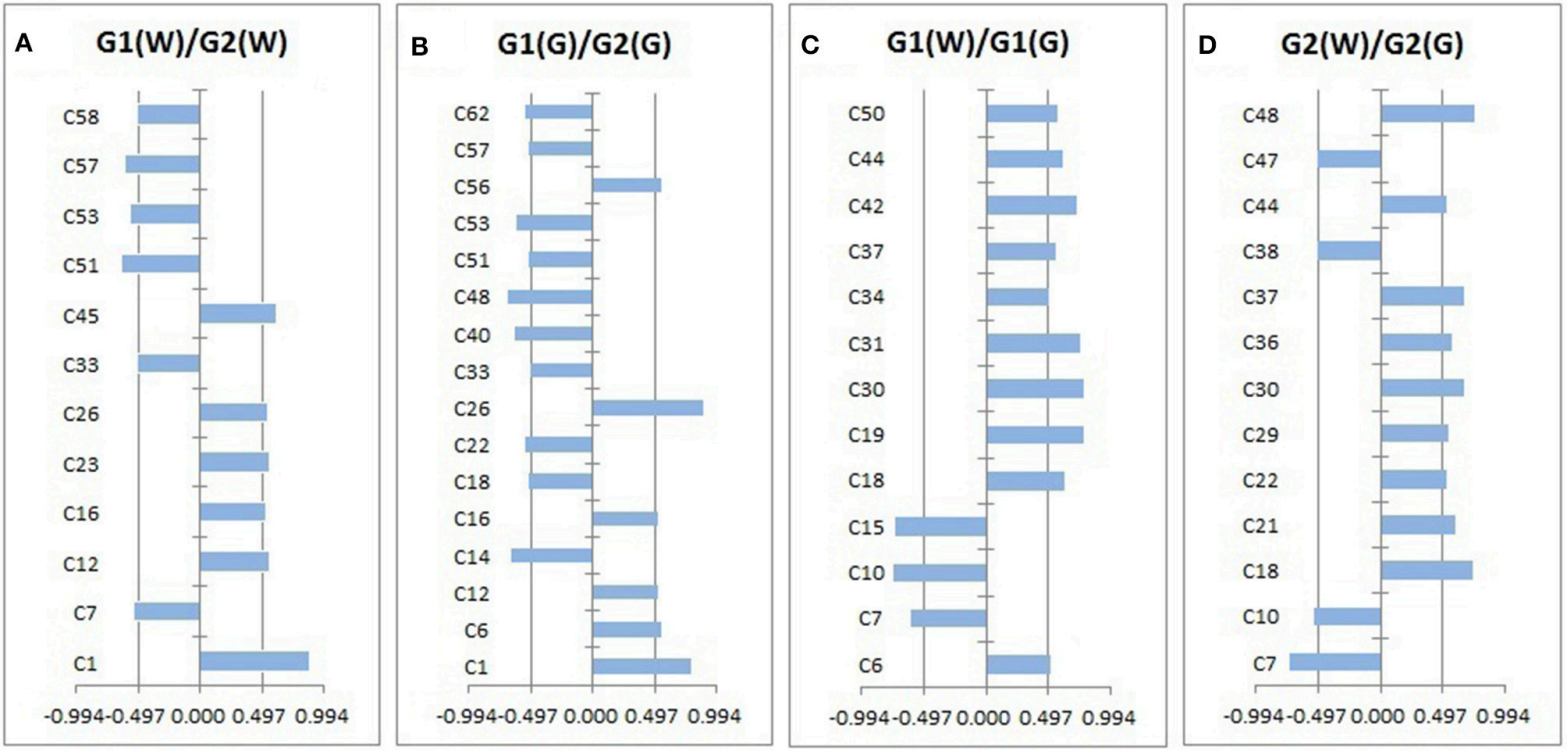
The volatile compounds with absolute values of correlation coefficients larger than 0.497 (i.e., significant intergroup differences).

Loadings plots from OPLS-DA (Figure 4, Table 4) showed all volatile compounds having significant inter-group differences between two genotype populations from wild and common garden samples respectively as well as between wild and common garden samples for G1 and G2, respectively. It is worth-noting that common chemotypic features can also be observed in both cases. For both wild and common garden samples, intergroup chemotypic differences are highlighted by significant higher levels of C1 (unknown), C12 (2,4(10)-thujadiene), C16 (1,3,5-trimethylencycloheptan), and C26 (2-acetylpyrrole) but lower levels of C33 (2-ethoxyethyl 3-methylbutanoate), C51 (α-guaiene), C53 (eudesma-3,7(11)-diene), and C57 (2,6,10,10-tetramethylbicyclo[7.2.0]undeca-2,6-diene) in G2 than in G1 (Figures 4A,B). Therefore, these 8 compounds were employable to discriminate G1 and G2. This suggests that two genotypic populations, G1 and G2, have significant difference in their metabolisms with C12, C51, C53 and C57 derived probably from terpenoid pathway. Furthermore, for both G1 and G2, intergroup chemotypic differences are also highlighted by significant higher levels of C18 (isobutyl isovalerate), C30 (3-oxobutan-2-yl 2-methylbutanoate), C37 (unknown), and C44 (pentanoyl pentanoate) but lower levels of C7 (3-methylbut-2-enoic acid) and C10 (3-methylvaleric acid) in common garden samples than in wild samples (Figures 4C,D). These 6 compounds could be used to distinguish the wild populations and common-garden samples (Table 4). This indicates that for both G1 and G2 environmental changes (by transplanting from wild to common gardens) induce significantly in their metabolisms of fatty acids and branch-chain amino acids since these inter-group differential organic acids and esters are mostly β-oxidation metabolites of fatty acids and keto-acids from oxidation of amino acids (Schwab et al., 2008). Moreover, C50 ((–)-β-caryophyllene) has significantly higher levels in common garden samples of G1 than in wild samples of same genotype, indicating that transplantation (or environmental factor) has apparent effects on its (2E,6E)-farnesyl diphosphate metabolism. β-caryophyllene can selectively bind to the cannabinoid receptor type 1 (CB1) and (CB2) thus can be used in the treatment of inflammation, pain, osteoporosis, and atheroscelerosis (Gertsch et al., 2008). It is, therefore, expected that the above effects of genotype, transplantation, or environmental changes on the levels of these compounds may have profound influences on the therapeutic efficiency of this particular herb. Hence planting bases is ought to be selected appropriately for efficacy and quality control purposes.

**Table 4:** Ranking of the volatile compounds with VIP values larger than 1.00, absolute values of correlation coefficients larger than 0.497, and *P*-values less than 0.05.

**No**.	**G1(W)/G2(W)**			**G1(G)/G2(G)**			**G1(W)/G1(G)**			**G2(W)/G2(G)**		
	***P*-value**	**CCΔ**	**VIP**	***P*-value**	**CC**	**VIP**	***P*-value**	**CC**	**VIP**	***P*-value**	**CC**	**VIP**
1	0.00E+00	0.885	0.363	0.00E+00	0.787	0.329	7.75E-04	0.286	0.204	–	–	–
2	–	–	–	2.08E-02	−0.216	0.069	–	–	–	4.62E-04	0.282	0.101
3	3.26E-05	0.436	0.325	8.98E-01	−0.078	0.040	4.75E-03	−0.332	0.349	7.71E-03	0.262	0.167
4	–	–	–	6.21E-01	−0.025	0.015	1.39E-04	0.396	0.344	2.36E-07	0.382	0.241
5	2.34E-02	0.241	0.157	1.13E-03	0.360	0.191	5.51E-05	0.469	0.453	2.30E-08	0.312	0.200
6	–	–	–	7.56E-09	0.548	0.132	1.39E-04	0.507	0.248	–	–	–
7	1.32E-07	−0.534	1.451	2.52E-08	−0.398	0.654	1.14E-12	−0.601	2.004	0.00E+00	−0.750	1.972
8	–	–	–	8.72E-03	−0.228	0.125	–	–	–	7.60E-05	0.260	0.160
9	–	–	–	1.84E-04	−0.343	0.397	–	–	–	2.99E-08	0.439	0.572
10	4.44E-01	−0.191	1.054	2.08E-02	−0.180	0.450	5.47E-11	−0.738	4.076	3.07E-11	−0.533	2.742
11	1.73E-02	−0.241	0.417	1.84E-04	−0.200	0.712	–	–	–	2.53E-03	0.232	0.917
12	1.76E-08	0.565	0.667	7.56E-09	0.529	0.636	5.59E-01	0.140	0.352	–	–	–
13	–	–	–	2.38E-03	0.364	0.295	1.39E-04	0.460	0.709	4.62E-04	0.258	0.181
14	7.09E-07	−0.386	0.346	6.66E-16	−0.656	0.570	–	–	–	4.99E-11	0.487	0.493
15	1.58E-01	−0.044	0.045	–	–	–	5.47E-11	−0.722	0.755	0.00E+00	−0.393	0.357
16	1.76E-08	0.524	0.307	1.31E-07	0.521	0.306	2.87E-04	0.373	0.435	2.36E-07	0.398	0.202
17	6.01E-01	0.250	0.229	–	–	–	1.39E-04	−0.454	0.598	2.81E-12	−0.406	0.277
18	–	–	–	9.13E-09	−0.519	0.460	1.85E-09	0.631	0.580	0.00E+00	0.737	0.802
19	1.73E-02	−0.221	0.199	5.75E-03	0.298	0.229	1.14E-12	0.770	1.100	1.29E-08	0.360	0.342
20	–	–	–	1.84E-04	−0.327	0.183	–	–	–	2.99E-08	0.411	0.258
21	1.02E-01	0.162	0.117	2.02E-05	−0.385	0.286	3.44E-01	0.127	0.132	9.53E-14	0.593	0.535
22	2.79E-06	−0.381	0.289	6.68E-14	−0.540	0.549	–	–	–	2.01E-12	0.525	0.606
23	1.76E-08	0.553	0.286	–	–	–	1.39E-04	−0.474	0.391	–	–	–
24	1.62E-01	−0.161	0.099	2.28E-01	−0.035	0.023	3.33E-02	0.313	0.370	5.19E-04	0.351	0.247
25	–	–	–	2.08E-02	−0.208	0.107	–	–	–	4.62E-04	0.272	0.156
26	1.76E-08	0.544	0.477	0.00E+00	0.887	0.740	1.10E-04	0.472	0.767	–	–	–
27	1.76E-08	0.485	0.241	–	–	–	1.39E-04	−0.380	0.303	–	–	–
28	–	–	–	2.08E-02	−0.209	0.101	–	–	–	4.62E-04	0.287	0.155
29	2.35E-09	−0.360	0.579	1.96E-09	−0.436	1.729	5.47E-11	0.451	1.058	0.00E+00	0.543	2.469
30	1.67E-07	−0.402	0.235	2.36E-05	−0.436	0.265	1.14E-12	0.776	0.735	0.00E+00	0.668	0.522
31	4.23E-02	−0.197	0.487	6.52E-01	−0.047	0.117	0.00E+00	0.753	2.719	2.55E-07	0.358	1.072
32	1.73E-02	−0.257	0.987	–	–	–	–	–	–	2.16E-04	−0.323	1.083
33	1.25E-08	−0.507	1.126	1.10E-07	−0.503	0.912	–	–	–	6.34E-01	0.086	0.188
34	1.62E-04	−0.342	0.366	1.25E-02	0.171	0.116	1.39E-04	0.500	0.570	1.57E-03	−0.284	0.278
35	–	–	–	2.08E-02	−0.226	0.204	–	–	–	4.62E-04	0.272	0.274
36	–	–	–	1.02E-09	−0.437	0.640	–	–	–	0.00E+00	0.564	0.943
37	–	–	–	6.46E-02	−0.180	0.223	1.85E-09	0.555	1.105	0.00E+00	0.670	0.973
38	2.38E-02	−0.260	0.290	–	–	–	1.39E-04	−0.487	0.456	1.93E-13	−0.507	0.508
39	8.35E-01	0.079	0.061	4.16E-03	−0.306	0.187	8.91E-02	0.201	0.198	4.35E-07	0.413	0.323
40	1.32E-07	−0.417	1.348	6.51E-14	−0.625	2.029	9.88E-01	−0.070	0.187	1.78E-08	0.452	1.668
41	–	–	–	2.08E-02	−0.198	0.109	–	–	–	4.62E-04	0.270	0.165
42	1.73E-02	−0.241	0.214	6.28E-01	0.051	0.050	1.85E-09	0.718	1.116	2.80E-06	0.384	0.434
43	–	–	–	8.72E-03	−0.213	0.113	–	–	–	7.60E-05	0.309	0.182
44	–	–	–	1.61E-03	0.343	0.741	5.47E-11	0.608	2.537	2.76E-13	0.523	1.089
45	7.79E-09	0.614	0.873	3.24E-04	0.266	0.389	1.60E-01	0.194	0.358	7.39E-01	0.176	0.289
46	3.65E-06	−0.430	1.088	7.79E-11	−0.477	1.444	1.87E-02	0.319	0.731	1.97E-08	0.425	1.461
47	3.48E-05	−0.375	0.471	–	–	–	–	–	–	4.41E-10	−0.497	0.552
48	–	–	–	6.68E-14	−0.675	0.833	–	–	–	0.00E+00	0.746	1.087
49	–	–	–	1.96E-01	−0.130	0.265	1.39E-04	0.412	0.787	7.60E-05	0.307	0.697
50	7.72E-02	0.060	0.238	2.66E-04	0.201	0.788	4.62E-04	0.570	3.600	9.29E-04	0.262	1.182
51	1.70E-11	−0.637	2.253	4.65E-13	−0.513	2.076	3.70E-02	0.217	0.652	3.33E-04	0.347	1.542
52				1.90E-01	−0.141	0.420	1.39E-04	0.403	1.059	7.60E-05	0.305	1.013
53	1.84E-08	−0.566	2.993	3.03E-14	−0.611	3.459	2.98E-02	−0.339	1.584	5.74E-04	0.341	2.091
54	1.38E-02	−0.308	0.753	4.13E-10	−0.448	1.296	1.56E-01	0.253	0.581	2.92E-07	0.416	1.382
55	3.06E-05	−0.430	1.283	1.76E-11	−0.429	1.794	7.13E-02	−0.243	0.764	4.04E-05	0.354	1.635
56	–	–	–	7.56E-09	0.547	0.327	1.39E-04	0.448	0.546	–	–	–
57	1.32E-10	−0.608	1.911	2.48E-14	−0.514	2.007	5.37E-01	−0.175	0.506	4.11E-05	0.357	1.519
58	8.39E-08	−0.502	2.764	8.67E-13	−0.492	3.382	8.53E-01	0.066	0.333	8.50E-07	0.403	3.082
59	1.02E-03	−0.358	0.828	4.09E-09	−0.354	1.282	3.08E-01	−0.127	0.319	5.03E-05	0.334	1.348
60	4.23E-02	−0.187	0.201	5.17E-04	−0.215	0.661	–	–	–	1.45E-03	0.286	0.980
61	–	–	–	2.08E-02	−0.185	0.156	–	–	–	4.62E-04	0.237	0.223
62	1.11E-04	−0.384	0.825	4.26E-12	−0.542	1.211	–	–	–	8.86E-07	0.397	1.023
63	–	–	–	8.72E-03	–	0.233	–	–	–	7.60E-05	0.311	0.354
64	–	–	–	2.08E-02	−0.165	0.189	–	–	–	4.62E-04	0.255	0.323

*CCΔ, Correlation coefficients; –, No detected in both of two groups; P-value = 0.00E + 00: No detected in one of two groups*.

Taking all these together, it is apparent that the chemotypic nature of volatile components in this plant depends on both genotypes and environmental factors with the present of two major genotypic populations. These will be expected to be vital information for understanding physiology of *V. jatamansi* and for potentially effective commercial applications.

## Conclusion

Two genotypicaly different groups were found for *V. jatamansi* based on genetic analysis and the volatile chemotypes of such groups were not only dependent upon their genotypic features but also environmental factors. When transplanted in common gardens (having similar environment), the two genotypic groups had their own unique composition of volatile constituents. Such genotypic and environmental factors appear to affect the plant secondary metabolites in terpenoid pathway and oxidation metabolism of fatty acids and amino acids.

Common chemotypic differences between two genotypic populations were identifiable for both wild and common garden samples. Eight volatile compounds were discriminatory compounds to differentiate two genotypes in both wild and/or common garden samples. Common chemotypic differences between wild and common garden samples were also observed for both genotypic groups. Six volatile compounds were selectable as discriminatory compounds to differentiate wild and common garden samples for both genotypes. These findings offered better understanding of the correlation of genotype and chemotype for *V. jatamansi* as well as its genetic and chemical variations in different regions and indicated that both genotyping and chemotyping may need to be considered in breeding new genotypic plants even though the current results themselves may offer little direct guidance at this stage. Nevertheless, selection of good germplasms and proper planting locations of *V. jatamansi* still need more research come with pharmacodynamic evaluation. It is conceivable that whole metabolomics phenotypes (apart from volatile chemotype) will be of importance for both plant physiology and potential commercial applications.

## Author contributions

HX conceived the original project and research plans. XH and ZY collected the samples. XH, SW, and ZS performed experiments. SW, JS, and ZL analyzed the data. HX, SW, and HT wrote paper with all authors participated. All authors agree to be accountable for the content of the work.

### Conflict of interest statement

The authors declare that the research was conducted in the absence of any commercial or financial relationships that could be construed as a potential conflict of interest.

## Supplementary material

Supplemental Figure 1 Map of the population location. Green marks show wild population, red mark show common-garden.

Supplemental Table 1 Volatile compounds identified by automatic retrieval of the mass spectra (NIST 14 and NIST 14s), assisted by retention index.

Supplemental Table 2 Volatile compounds in the populations.
