# Supplementary figures and images for: Genotypic and Environmental Effects on the Volatile Chemotype of Valeriana jatamansi Jones
Source: Front Plant Sci. 2018 Jul 10;9:1003. doi: 10.3389/fpls.2018.01003 (PMC6048435; doi:10.3389/fpls.2018.01003)

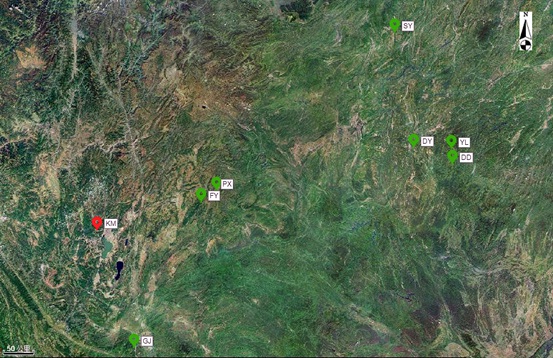

Supplement: Supplemental Figure 1 — Map of the population location. Green marks show wild population, red mark show common-garden. [file Image_1.JPEG]
