# Supplementary material for: Genotypic and Environmental Effects on the Volatile Chemotype of Valeriana jatamansi Jones
Source: Front Plant Sci. 2018 Jul 10;9:1003. doi: 10.3389/fpls.2018.01003 (PMC6048435; doi:10.3389/fpls.2018.01003)
Supplement: Supplemental Table 1 — Volatile compounds identified by automatic retrieval of the mass spectra (NIST 14 and NIST 14s), assisted by retention index. [file Table_1.DOCX]

**Supplemental Table 1.** Volatile compounds identified by automatic retrieval of the mass spectra (NIST 14 and NIST 14s), assisted by retention index.

| **NO.** | Compound Name | **Similarity** | **Ret.Index** | **RI*** | **△RI** | **Formula** | **Classification** | **CAS** | **Library** |
| --- | --- | --- | --- | --- | --- | --- | --- | --- | --- |
| 1 | unknown | 87 | 756 | - | -9 | - | - | - | - |
| 2 | 3-methylpentanal | 94 | 767 | 742 | 25 | C_6_H_12_O | Aldehydes | 15877-57-3 | NIST14s |
| 3 | Methyl Isovalerate | 94 | 774 | 774 | 0 | C_6_H_12_O_2_ | Esters | 556-24-1 | NIST14s |
| 4 | Propyl propionate | 83 | 785 | 785 | 0 | C_6_H_12_O_2_ | Esters | 106-36-5 | NIST14s |
| 5 | Hexanal | 97 | 797 | 806 | -9 | C_6_H_12_O | alkene | 66-25-1 | NIST14s |
| 6 | 2,4,4-Trimethyl-1-hexene | 85 | 818 | 799 | 19 | C_9_H_18_ | Acids | 51174-12-0 | NIST14s |
| 7 | 3-methylbut-2-enoic acid | 94 | 915 | 860 | 55 | C_5_H_8_O_2_ | Acids | 541-47-9 | NIST14 |
| 8 | Bicyclo[3.1.0]hex-2-ene, 2-methyl-5-(1-methylethyl)- | 95 | 931 | 902 | 29 | C_10_H_16_ | Monoterpenes | 2867-5-2 | NIST14s |
| 9 | (+)-α-pinene | 90 | 941 | 948 | -7 | C_10_H_16_ | Monoterpenes | 7785-70-8 | NIST14 |
|  | α-Pinene | 90 | 941 | 948 | -7 | C_10_H_16_ | Monoterpenes | 80-56-8 | NIST14s |
|  | (1S)-2,6,6-Trimethylbicyclo[3.1.1]hept-2-ene | 89 | 941 | 948 | -7 | C_10_H_16_ | Monoterpenes | 7785-26-4 | NIST14 |
| 10 | DL-3-Methylvaleric acid | 96 | 948 | 910 | 38 | C_6_H_12_O_2_ | Acids | 105-43-1 | NIST14 |
| 11 | Camphene | 91 | 957 | 943 | 14 | C_10_H_16_ | Monoterpenes | 79-92-5 | NIST14s |
|  | Bicyclo[2.2.1]heptane, 2,2-dimethyl-3-methylene-, (1S)- | 91 | 957 | 943 | 14 | C_10_H_16_ | Monoterpenes | 5794-4-7 | NIST14 |
| 12 | 2,4(10)-thujadiene | 93 | 962 | 879 | 83 | C_10_H_14_ | Monoterpenes | 36262-09-6 | NIST14s |
| 13 | sabinene | 94 | 980 | 897 | 83 | C_10_H_16_ | Monoterpenes | 3387-41-5 | NIST14s |
| 14 | β-pinene | 95 | 987 | 943 | 44 | C_10_H_16_ | Monoterpenes | 127-91-3 | NIST14s |
|  | (-)-β-pinene | 95 | 987 | 943 | 44 | C_10_H_16_ | Monoterpenes | 18172-67-3 | NIST14 |
| 15 | 2-Pentylfuran | 95 | 993 | 1040 | -47 | C_9_H_14_O | heterocycles | 3777-69-3 | NIST14 |
| 16 | 1,3,5-Trimethylencycloheptan | 90 | 1000 | 1039 | -39 | C_10_H_14_ | - | 68284-24-2 | NIST14 |
| 17 | unknown | - | 1003 | - | - | - | - | - | - |
| 18 | Isobutyl Isovalerate | 91 | 1006 | 955 | 51 | C_9_H_18_O_2_ | Esters | 589-59-3 | NIST14 |
| 19 | (3E,5E)-2,6-Dimethyl-1,3,5,7-octatetrene | 87 | 1011 | 966 | 45 | C_10_H_14_ | Monoterpenes | 460-01-5 | NIST14 |
| 20 | α-terpinene | 94 | 1024 | 998 | 26 | C_10_H_16_ | Monoterpenes | 99-86-5 | NIST14 |
| 21 | o-Cymene | 95 | 1031 | 1042 | -11 | C_10_H_14_ | Aromatics | 527-84-4 | NIST14s |
|  | p-Cymene | 94 | 1031 | 1042 | -11 | C_10_H_14_ | Aromatics | 99-87-6 | NIST14s |
|  | 1-methyl-3-propan-2-ylbenzene | 94 | 1031 | 1042 | -11 | C_10_H_14_ | Aromatics | 535-77-3 | NIST14s |
| 22 | (4R)-limonene | 94 | 1036 | 1018 | 18 | C_10_H_16_ | Monoterpenes | 5989-27-5 | NIST14 |
|  | Limonene | 93 | 1036 | 1018 | 18 | C_10_H_16_ | Monoterpenes | 138-86-3 | NIST14s |
| 23 | 4-Methyl-2-oxovaleric acid | 87 | 1046 | 1046 | 0 | C_6_H_10_O_3_ | Acids | 816-66-0 | NIST14 |
| 24 | phenylacetaldehyde | 96 | 1051 | 1081 | -30 | C_8_H_8_O | Aromatics | 122-78-1 | NIST14s |
| 25 | γ-terpinene | 97 | 1065 | 998 | 67 | C_10_H_16_ | Monoterpenes | 99-85-4 | NIST14 |
| 26 | 2-acetylpyrrole | 94 | 1069 | 1035 | 34 | C_6_H_7_NO | Nitrogen organic compounds | 1072-83-9 | NIST14s |
| 27 | 3-ethyl-2,5-dimethylpyrazine | 94 | 1084 | 1107 | -23 | C_8_H_12_N_2_ | Nitrogen organic compounds | 13360-65-1 | NIST14s |
| 28 | terpinolene | 91 | 1097 | 1052 | 45 | C_10_H_16_ | Monoterpenes | 586-62-9 | NIST14 |
| 29 | isoamyl valerate | 91 | 1105 | 1118 | -13 | C_10_H_20_O_2_ | Esters | 2050-9-1 | NIST14s |
| 30 | 3-Oxobutan-2-yl 2-methylbutanoate | 91 | 1116 | 1091 | 25 | C_9_H_16_O_3_ | - | - | NIST14 |
| 31 | Unknown | - | 1127 | - | - | - | - | - | - |
| 32 | 3-hydroxyisovaleric acid | 85 | 1133 | 966 | 167 | C_5_H_10_O_3_ | Acids | 625-08-1 | NIST14s |
| 33 | 2-Ethoxyethyl 3-methylbutanoate | 83 | 1158 | 1095 | 63 | C_9_H_18_O_3_ | - | - | NIST14 |
| 34 | Isovaleric Anhydride | 87 | 1187 | 1190 | -3 | C_10_H_18_O_3_ | anhydrides | 1468-39-9 | NIST14s |
| 35 | Unknown | - | 1208 | - | - | - | - | - | - |
| 36 | 2-methoxy-4-methyl-1-propan-2-ylbenzene | 89 | 1235 | 1231 | 4 | C_11_H_16_O | Aromatics | 1076-56-8 | NIST14 |
| 37 | Unknown | - | 1240 | - | - | - | - | - | - |
| 38 | Unknown | - | 1240 | - | - | - | - | - | - |
| 39 | Unknown | - | 1283 | - | - | - | - | - | - |
| 40 | Bornyl acetate | 97 | 1299 | 1277 | 22 | C_12_H_20_O_2_ | Esters | 76-49-3 | NIST14s |
|  | (1R,4S)-1,7,7-trimethylbicyclo[2.2.1]heptan-2-yl acetate | 97 | 1299 | 1277 | 22 | C_12_H_20_O_2_ | Esters | 92618-89-8 | NIST14s |
|  | Isobornyl acetate | 94 | 1299 | 1277 | 22 | C_12_H_20_O_2_ | Esters | 125-12-2 | NIST14 |
| 41 | Unknown | - | 1307 | - | - | - | - | - | - |
| 42 | 3-methyl-2-(3-methylbut-2-enyl)furan | 88 | 1326 | 1293 | 33 | C_9_H_10_O_2_ | heterocycles | 15186-51-3 | NIST14 |
| 43 | Terpinyl Acetate | 95 | 1355 | 1333 | 22 | C_12_H_20_O_2_ | Acids | 80-26-2 | NIST14s |
| 44 | pentanoyl pentanoate | 88 | 1387 | 1319 | 68 | C_10_H_18_O_3_ | anhydrides | 2082-59-9 | NIST14s |
| 45 | (-)-α-CUBEBENE | 91 | 1396 | 1344 | 52 | C_15_H_24_ | Sesquiterpenoids | 17699-14-8 | NIST14s |
| 46 | (-)-β-Elemene | 94 | 1407 | 1398 | 9 | C_15_H_24_ | Sesquiterpenoids | 515-13-9 | NIST14s |
| 47 | 1H-Cyclopropa[a]naphthalene, decahydro-1,1,3a-trimethyl-7-methylene-, [1aS-(1a.alpha.,3a.alpha.,7a.beta.,7b.alpha.)]- | 85 | 1424 | 1386 | 38 | C_15_H_24_ | - | 20071-49-2 | NIST14 |
| 48 | 1,4-dimethoxy-2-methyl-5-propan-2-ylbenzene | 92 | 1424 | - | - | C_12_H_18_O_2_ | Aromatics | 14753-08-3 | NIST14 |
| 49 | alpha-santalene | 96 | 1432 | 1211 | 221 | C_15_H_24_ | Sesquiterpenoids | 512-61-8 | NIST14 |
| 50 | (-)-β-caryophyllene | 96 | 1440 | 1494 | -54 | C_15_H_24_ | Sesquiterpenoids | 87-44-5 | NIST14s |
|  | (1R,4Z,9S)-4,11,11-trimethyl-8-methylidenebicyclo[7.2.0]undec-4-ene | 95 | 1440 | 1494 | -54 | C_15_H_24_ | Sesquiterpenoids | 118-65-0 | NIST14s |
| 51 | α-guaiene | 98 | 1451 | 1490 | -39 | C_15_H_24_ | Sesquiterpenoids | 3691-12-1 | NIST14 |
|  | Aciphyllene | 90 | 1451 | 1490 | -39 | C_15_H_24_ | Sesquiterpenoids | 87745-31-1 | NIST14 |
| 52 | (S)-β-bisabolene | 88 | 1457 | 1500 | -43 | C_15_H_24_ | Sesquiterpenoids | 495-61-4 | NIST14s |
| 53 | Eudesma-3,7(11)-diene | 89 | 1469 | 1507 | -38 | C_15_H_24_ | Sesquiterpenoids | 6813-21-4 | NIST14 |
| 54 | α-Humulene | 94 | 1474 | 1579 | -105 | C_15_H_24_ | Sesquiterpenoids | 6753-98-6 | NIST14 |
| 55 | α-patchoulene |  | 1483 | 1403 | 80 | C_15_H_24_ | - | 560-32-7 | NIST14s |
| 56 | (+)-γ-GURJUNENE | 97 | 1501 | 1461 | 40 | C_15_H_24_ | Sesquiterpenoids | 22567-17-5 | NIST14s |
| 57 | 2,6,10,10-Tetramethylbicyclo[7.2.0]undeca-2,6-diene | 88 | 1516 | 1499 | 17 | C_15_H_24_ | - | 136296-37-2 | NIST14s |
| 58 | alpha-Bulnesene | 91 | 1521 | 1490 | 31 | C_15_H_24_ | Sesquiterpenoids | 3691-11-0 | NIST14s |
| 59 | α-panasinsanene | 97 | 1536 | 1416 | 120 | C_15_H_24_ | Sesquiterpenoids | 56633-28-4 | NIST14 |
| 60 | Kessane | 94 | 1545 | - | - | C_15_H_26_O | - | 3321-66-2 | NIST14 |
| 61 | hedycaryol | 92 | 1559 | 1694 | -135 | C_15_H_26_O | Sesquiterpenoids | 21657-90-9 | NIST14 |
|  | Elemol | 96 | 1559 | 1522 | 37 | C_15_H_26_O | Sesquiterpenoids | 639-99-6 | NIST14s |
| 62 | pogostol | 94 | 1679 | - | - | C_15_H_26_O | Sesquiterpenoids | 21698-41-9 | NIST14 |
| 63 | Longifolenaldehyde | 91 | 1701 | 1581 | 120 | C_15_H_24_O | - | 19890-84-7 | NIST14 |
| 64 | unknown | 87 | 1744 | - | - | - | - | - | - |

**RI*:** Ret.Index in the librarys.
